# Identification of the Bisabolol Synthase in the Endangered Candeia Tree (*Eremanthus erythropappus* (DC) McLeisch)

**DOI:** 10.3389/fpls.2018.01340

**Published:** 2018-09-19

**Authors:** Leticia Alves Gomes Albertti, Thierry L. Delatte, Katyuce Souza de Farias, Amanda Galdi Boaretto, Francel Verstappen, Adele van Houwelingen, Katarina Cankar, Carlos Alexandre Carollo, Harro J. Bouwmeester, Jules Beekwilder

**Affiliations:** ^1^Laboratório de Evolução e Biodiversidade Evolutiva, Universidade Federal de Mato Grosso do Sul, Campo Grande, Brazil; ^2^Laboratory of Plant Physiology, Wageningen University & Research, Wageningen, Netherlands; ^3^Laboratório Productos Natural & Espectrometria Massas, Universidade Federal de Mato Grosso do Sul, Campo Grande, Brazil; ^4^Wageningen Plant Research, Wageningen, Netherlands

**Keywords:** (–)-α-bisabolol, *Eremanthus erythropappus* (DC) McLeisch, Candeia, sesquiterpene, bisabolol synthase

## Abstract

Candeia (*Eremanthus erythropappus* (DC) McLeisch, Asteraceae) is a Brazilian tree, mainly occurring in the cerrado areas. From ethnobotanical information its essential oil is known to have wound healing and nociceptive properties. These properties are ascribed to result from a sesquiterpene alcohol, (–)-α-bisabolol, which is present at high concentrations in this oil. Bisabolol is highly valued by the cosmetic industry because of its antibacterial, anti-inflammatory, skin-smoothing and wound healing properties. Over the past decades, Candeia timber has been collected at large scale for bisabolol extraction from wild reserves and the species is thereby at risk of extinction. To support the development of breeding and nursing practices that would facilitate sustainable cultivation of Candeia, we identified a terpene synthase gene, EeBOS1, that appears to control biosynthesis (–)-α-bisabolol in the plant. Expression of this gene in *E. coli* showed that EeBOS1 protein is capable of producing (–)-α-bisabolol from farnesyl pyrophosphate *in vitro*. Analysis of gene expression in different tissues from Candeia plants in different life stages showed a high correlation of EeBOS1 expression and accumulation of (–)-α-bisabolol. This work is the first step to unravel the pathway toward (–)-α-bisabolol in Candeia, and in the further study of the control of (–)-α-bisabolol production.

## Introduction

**FIGURE 1:**
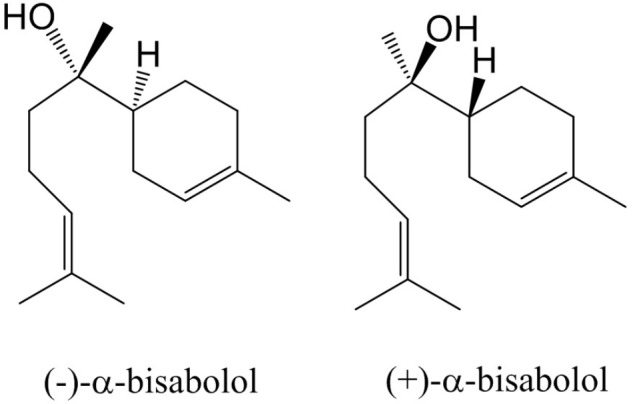
Structures of α-bisabolol isomers.

Essential oils from wild plants have been applied for many centuries in traditional medicine (Kamatou and Viljoen, 2010). In recent years, knowledge from indigenous people is being explored to scout for novel medicines. In particular, in areas that are rich in biodiversity, such as the Brazilian Cerrado, ethnobotanists have scouted for plants whose extracts or oils have potential as pharmaceuticals (Ferreira-Rodrigues et al., 2016; Borges et al., 2017; Oliveira et al., 2017). One plant that has been identified as a source of high-value essential oil is the Candeia tree (*Eremanthus erythropappus* (DC) McLeisch), which primarily grows in the state of Minas Gerais in Brazil. The oil which is steam distilled from trunks of this plants is known for its wound-healing, antinociceptive and anti-inflammatory properties (Sousa et al., 2008). A dominant active ingredient in Candeia oil is (–)-α-bisabolol, which is a monocyclic sesquiterpene alcohol (**Figure 1**). Its activities include anti-inflammatory (Kim et al., 2011), antifungal, antibacterial (De Lucca et al., 2011), gastro protective (Bezerra et al., 2009), and anti-cancer effects (Cavalieri et al., 2004; Da Silva et al., 2010; Seki et al., 2011). Due to its wound-healing and skin permeation enhancing effects it is frequently used as an additive to skin care products such as balms and aftershaves 1. (–)-α bisabolol was first described as an active component of German chamomile (*Matricaria recutita* L.) (Mckay and Blumberg, 2006; Kamatou and Viljoen, 2010). Four stereo isomers have been found in other plant species, due to the presence of two chiral centers (Gunther et al., 1993). The most bio-active form of bisabolol is the (–)-α-Bisabolol, with the (4S, 8S)-configuration. Chemical synthesis of bisabolol results in racemic mixtures, which have a lower bio-activity compared to the Candeia oil ingredient (Schwartz and Swanson, 1979). Candeia wood essential oil was reported to be dominated by (–)-α-bisabolol, up to a level of 66 to 91%. In leaves, (–)-α-bisabolol constitutes 2–24% of the oil (Silverio et al., 2013; Dos Santos et al., 2015).

Until recently, most of the (–)-α-bisabolol on the world market world is produced from Candeia trees (De Oliveira et al., 2009). In the period from 1980 to 2010, up to 250 tons of Candeia oil were harvested (De Oliveira et al., 2010), which is predominantly collected by grubbing trees from the wild. Economic quantities of (–)-α-bisabolol can only be distilled from trees older than 10 years implying the risk of deforestation for oil extraction (Silva et al., 2012). As an alternative to collection from the wild, commercial exploitation of Candeia plantation would be an attractive solution for the production of high quality timber and natural (–)-α-bisabolol (Silva et al., 2012; Scolforo et al., 2016).

To sustain the annual oil production, at least 30,000 m^3^ of Candeia wood is needed, equivalent to approximately 1000 ha of forest. However, cultivation of Candeia is not straightforward, long-term strategies are needed and Candeia germplasm with predictable productivity after 10 years is not yet available. Therefore, illegal logging of wild material is still continuing, and will eventually cause an ecological imbalance, even putting this species at risk of extinction (Silva et al., 2012). To support such a commercial exploitation there is a need for a better understanding of the physiological processes leading to the biosynthesis of (–)-α-bisabolol (Mori et al., 2010), and the underlying biosynthetic genes. Since the relevant level of bisabolol in trunks can only be quantified after 10 years, understanding the bisabolol pathway and regulation of the bisabolol level would strongly enhance Candeia breeding practices.

In this work, we take the first step toward identification of a genetic marker, by identifying a Candeia gene encoding an (–)-α-bisabolol synthase that produces (–)-α-bisabolol as a single product and correlate the gene expression with the (–)-α-bisabolol accumulation *in planta*.

## Materials and Methods

### Plant Material

Plant material from *E. erythropappus* was collected from the nursery of professor L. Amaral de Melo (Departamento de Ciências Florestais, Laboratório de Silvicultura) at the Universidade Federal de Lavras (Lavras, MG - Brazil - Caixa-Postal: 3037). The samples were collected from genetically identical plants from different ages. Materials from *Lippia dulcis* and *Nicotiana benthamiana* expressing *Artemisia* bisabolol synthase were obtained from previous studies (Yang et al., 2011; Delatte et al., 2018).

### Extraction and Isolation

All chemicals were purchased at Sigma-Aldrich if not otherwise indicated. Plant material (0.1 g) was weighed in a precooled glass tube, and extracted with 1 mL of dichloromethane. Samples from hard tissues were first frozen in liquid nitrogen then broken in pieces with a hammer before being pulverized with a grinding mill (IKA type A11, VWR). The suspension was vortexed (1 min), then sonicated (15 min, Branson 3510) and centrifuged for 5 min at 1500 *g* at room temperature. The supernatant was collected, dehydrated using a column of 1 g sodium sulfate and analyzed on GC/MS. Annotation was performed with retention indices, mass spectra and authentic standards. Quantification was performed with an external calibration curve from (–)-α-bisabolol.

### Gas Chromatography

Chemical analysis was performed on an Agilent 7890A gas chromatograph connected to a 5975C mass selective Triple-Axis Detector (Agilent Technologies). For quantification of bisabolol, 1 μL of each sample was injected at 250°C in split-less mode on a ZB-5MS column (Zebron, Phenomenex, 30 m × 250 μm × 0.25 μm film thickness) with 5 m guard column, with a constant flow of helium at 1 mL/min. For chiral analysis the same GC-MS set up was used, using an Alpha DEX^TM^ 120 (Supelco; 30 m × 0.25 mm × 0.25 μm) column. With the ZB-5MS the oven was programmed for 1 min at 45°C, then ramped at 10°C/min to 300°C and kept as such for 5 min with a solvent delay of 5.5 min, for a final run time of 31.5 min. With the Alpha DEX^TM^, the oven was programmed for 1 min at 50°C, then ramped at 2°C/min to 175°C and then ramped at 10°C/min to 190°C kept as such for 1 min with a solvent delay of 5 min, for a final run time of 66 min. The ionization potential was set at 70 eV, and scanning was performed from 45 to 400 atomic mass units, with a scanning speed of 3.99 scans/s. Samples from Candeia trees were separated in branch (*n* = 5), leaf (*n* = 3), and root (*n* = 4).

### RNA Isolation and Sequence Analysis

RNA isolation was performed with a hot borate method (Wan and Wilkins, 1994). Wood samples were frozen in liquid nitrogen before being broken in pieces with a hammer then pulverized with a grinding mill (IKA type A11). Around 70–90 mg of ground material was extracted with 800 μL extraction buffer preheated at 80°C [0.2 M sodium borate, pH 9.0; 30 mM EGTA; 1% (w/v) sodium dodecyl sulfate; 1% (w/v) sodium deoxycholate, 6% (w/v) polyvinylpyrrolidinone K 40, 10 mM dithiothreitol], and incubated at 42°C for 15 min, after which samples were centrifuged (4°C, 12000 *g*) for 20 min. The supernatant was transferred to new tubes and 260 μL of ice-cold 8 M lithium chloride (LiCl) was added before a 4°C over-night incubation. After centrifugation (4°C, 12000 *g*) for 30 min, the supernatant was discarded and 750 μL ice-cold 2 M LiCl was added. The tubes were centrifuged again (4°C, 12000 *g*, 10 min), the supernatant discarded and then the pellet was air-dried for 20 min. The dried pellet was dissolved in 150 μL DEPC treated water and extracted once with an equal volume of chloroform:isoamylalcohol (24:1, v:v). After centrifugation (4°C, 12000 *g*) for 10 min the aqueous phase was transferred to a new tube. The RNA was precipitated overnight at -20°C in the presence of sodium acetate (final centration 300 mM, pH 5.2), ethanol (2.5× volume, ice-cold) and glycogen (45 μg). The RNA was pelleted by centrifugation (4°C, 12000 *g*) for 30 min, and the pellet washed with 250 μL of ice-cold 70% ethanol (v/v). After 10 min of centrifugation (4°C, 12000 *g*) the pellet was air-dried and suspended in water (DEPC treated).

Total RNA was used to prepare a cDNA library using TruSeqTM RNA preparation kit (Illumina, United States) for Illumina HiSeq2000 at Vertis Biotechnology AG (Freising, Germany). Illumina HiSeq2000 sequencing resulted in 81,396,422 total reads between 36–251 bp long. Bisabolol synthases known from the literature, including *L. dulcis* (+)-epi-α-bisabolol synthase (J7LH11), *Artemisia annua* α-bisabolol synthase (4FJQ), *Matricaria* bisabolol synthase AIG92846 were blasted against Candeia transcriptome in the Trinity web tool, using TBLASTN with a cut-off *e*-value of 1e-4. After annotations and assembling, contigs were manually inspected to identify coding regions and full length open reading frames. The best hits were blasted on UniProt database Uni Prot Consortium (2017). Multiple protein sequence alignments were performed using the CLC Main Workbench (QIAGEN, Version 7.6.4).

### Cloning EeBOS1

The most likely candidate identify from Illumina the was extended to full-length cDNA sequences using the SMART RACE cDNA Amplification Kit from Clontech. Total RNA isolated from the branch was used to generate 3^′^RACE cDNA according to the Kit’s descriptions (**Supplementary Table 2**). The PCR products were verified by Sager sequencing (Macrogen Europe) before being cloned in pGEM Teasy vectors (Promega).

### Protein Expression and Enzymatic Assay

To obtain recombinant EeBOS, the ORFs of full-length EeBOS was cloned into pACYCDuet-1 vector (Novagen). Recombinant pACYCDuet-1:EeBOS construct was transformed into *E. coli* BL21 DE3 (Stratagene) and selected with chloramphenicol (50 μg⋅ml^-1^). A single transformed colony was cultured in LB medium with chloramphenicol (50 μg⋅ml^-1^) supplemented with 1% (W/V) glucose. When OD600 was between 0.6 and 0.8 the expression of EeBOS was then induced with 1 mM IPTG (final concentration) then the culture was incubated at 18°C overnight. The cells were harvested by centrifugation and immediately used for an *in vitro* enzyme assay.

The cells were re-suspended in buffer A [50 mM Tris/HCl (pH 7.5), 1.4 mM 2-mercaptoethanol]. After cell lysis by sonication and centrifugation (13000 *g*, 10 min, 4°C), the clear supernatant was used for enzymatic assay. Hundred microliters of the crude extract was diluted with 800 μl of buffer [15 mM MOPSO (pH 7.0), 12.5% (v/v) glycerol, 1 mM MgCl2, 1 mM ascorbic acid, 1 mM dithiothreitol, 5 mM sodium ortho-vanadate] and 10 mM farnesyl diphosphate (FPP) or geranyl diphosphate (GPP) as substrate. The mixture was covered with 1 mL of pentane layer and incubated at 30°C with mild agitation for 2 h, followed by extraction with 2 mL ethyl acetate (vortex-mixed and centrifuged at 1200 *g* for 10 min). The collected pentane layer was dried over a sodium sulfate column then analyzed by GC-MS.

### Quantitative RT PCR

Total RNA was used as template to synthesized cDNA with iScriptTM cDNA synthesis (Bio-Rad) according to the manufacturer’s instructions. After DNase treatment (Thermo Fisher Scientific), Q-PCR was performed using Power SYBR Green (Applied Biosystems) in a 10 μl reaction using the standard program of the CFX ConnectTM instrument (Bio-Rad). Data were analyzed using Bio-Rad CFX Manager version: 3.0.1215.0601 (Bio-Rad). The reference gene use for this study was obtained by homology to the *Chrysolaena obovata* elongation 1-α factor (KM597066). A Candeia elongation factor-encoding fragment was amplified from Candeia cDNA using primers designed on sequence of *C. obovata* which are conserved among asteraceae species (**Supplementary Table 2**). The product obtained was sequenced. Based on this sequence we designed a set of reference primers used in our study. All primers used are provided in the supporting information (**Supplementary Table 2**). Samples from Candeia trees were separated in branch (*n* = 5), leaf (*n* = 3), and root (*n* = 4).

### Accession Numbers

The nucleotide sequences of Candeia reported in this work have been submitted to the GenBank under the accession number MH048990 for the EeBOS and MH048991 for the EeEF1.

## Results

### Analysis of Candeia Material for Bisabolol

In order to determine relative quantities of (–)-α-bisabolol in different tissues, three Candeia plants which were genetically identical, but 1, 5, and 10 years old, were sampled. Dichloromethane (DCM) extracts from grinded material were analyzed by GC-MS using a non-chiral column for the presence of sesquiterpenes (**Supplementary Table 1**). This analysis was aimed to confirm the presence of bisabolol, and to investigate the proportion of bisabolol among DCM-extracted compounds. In none of the 1-year old sapling tissues any bisabolol could be detected, but a suite of other sesquiterpenes was observed, including (E)-β-caryophyllene and copaene. In the 5-year old tree, again these two sesquiterpenes were dominant, but also bisabolol could clearly be detected as a major peak in woody limb and twig samples, and to a lesser extent in root tissue. The proportion of bisabolol in the sesquiterpene mixture greatly increases in the 10 year-old samples, which was consistent with previously published research (Mori et al., 2010). In particular woody tissues such as limb and twig, but also in different root samples, high proportions of bisabolol were found. In the 10-year old tree samples, (E)-β-caryophyllene was only detected in leaf tissues.

**FIGURE 2:**
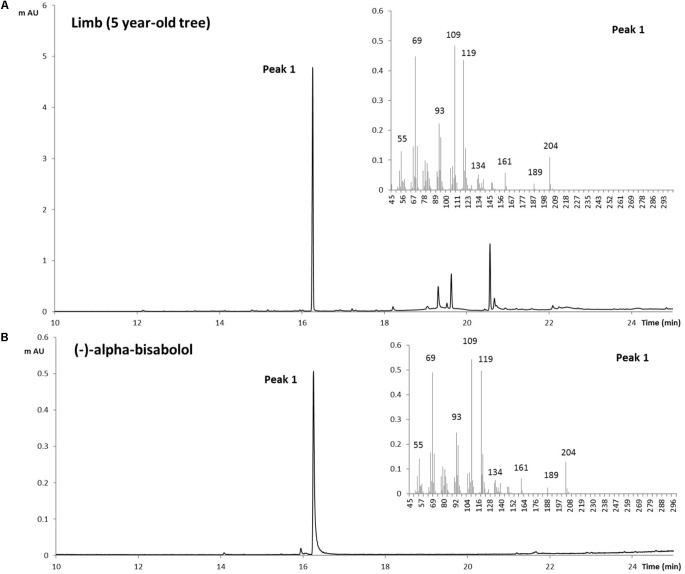
Identification of (–)-α-Bisabolol in Candeia. Extract from the limb of Candeia tree (5 years) was obtained with dichloromethane then analyzed by GC-MS **(A)**, and compared to a standard of (–)-α-Bisabolol **(B)**. On the right side of both panels are the MS spectra for the peak number 1.

**FIGURE 3:**
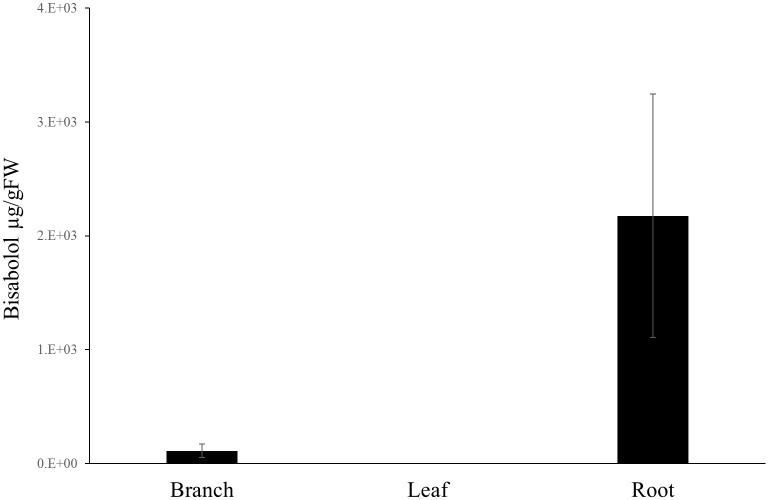
Bisabolol content in different tissues. (–)-α-Bisabolol was extracted with dichloromethane and quantified by GC-MS for different tissues originating from Candeia trees. Each bar is the average of at least three biological replicates ± SE.

To confirm the identification of α-bisabolol from the Candeia samples we compared the mass spectrum (**Figure 2A**) and retention index of the peak identified as α-bisabolol corresponded to an authentic (–)-α bisabolol standard (**Figure 2B**). Subsequently, α-bisabolol was quantified in different tissues from the Candeia tree, using an external calibration curve. As appears from **Figure 3** and **Supplementary Figure 3**, in particular the root material and the mature branches were found to be rich in bisabolol. The highest content of bisabolol was found in the lateral root samples of the 10-year old tree, at 10 mg/g fresh weight. Interestingly, this tissue also had a very high concentration of an unidentified diterpene (**Supplementary Table 1**). In leaves bisabolol could not be detected (**Figure 3** and **Supplementary Table 1**). The absence of bisabolol in this tissue was reported by Sousa et al. (2008), but differs from the results reported by Silverio et al. (2013), where α-bisabolol was detected in the leaves.

### Isolation of the (–)-α-Bisabolol Synthase From Candeia

Based on bisabolol quantification we chose to extract the RNA from the woody part of the branch of the 10 year old Candeia tree. Total RNA was extracted from the sample with hot borate method (Wan and Wilkins, 1994). The cDNA obtained from this RNA was analyzed by Illumina HiSeq2000 paired end sequencing using a read-length of about 250 bp. The sequencing generated 81.396.422 total sequence reads, from which 65.117.137 cleaned reads were used for sequence assembly. The assembly was probed for the presence of sesquiterpenes synthase-encoding sequences using TBLASTN, which resulted in 87 candidate terpene synthase transcripts (2017). Among these sequences, a transcript encoding a protein of 574 amino acids was identified, which showed 71% sequence identity to the recently identified (–)-α-bisabolol synthase from *M. recutita* (Son et al., 2014). This Candeia cDNA sequence was thereafter referred to EeTPS1.

### Identifying the Products of EeTPS1

**FIGURE 4:**
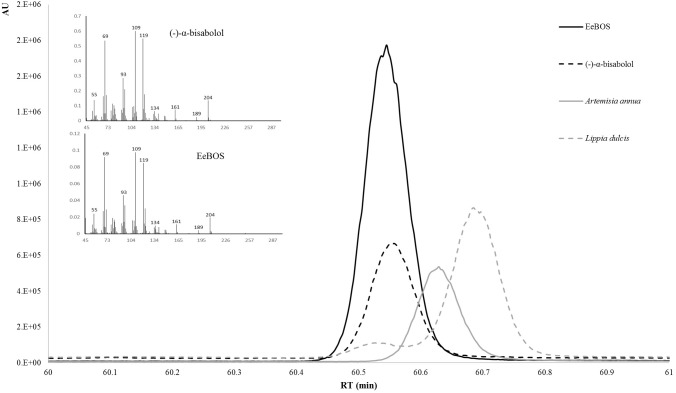
Enantioselective identification of the product of EeBOS. The product of the *in vitro* enzymatic assay with FPP were analyzed by GC-MS with a cyclodextrin column (solid black line). To confirm the enantiomer the EeBOS sample was compared with pure standards of [(–)-α-bisabolol; dotted black line], a known (+)-epi-bisabolol (*L. dulcis* extracts; dotted gray line), a known (+)-α-bisabolol (extract of *N. benthamiana* expressing *A. annua* bisabolol synthase; solid gray line). On the left side of both panels are the MS spectra for the (–)-α-bisabolol standard and EeBOS the *in vitro* enzymatic assay with FPP.

The full length open reading frame of EeTPS1 was amplified from Candeia cDNA and cloned into expression vector pACYCDUET-1 (**Supplementary Table 2**). The EeTPS1 protein was expressed in *E. coli* BL21 DE3, and cell-free extracts of EeTPS1 and pACYCDUET-1 hosting bacteria were compared in *in vitro* assays, using FPP or GPP as substrate. Assays of EeTPS1 with GPP did not show any product other than those detected using a control cell extract (**Supplementary Figure 1**). Assays with FPP did generate a single peak which showed the same mass spectrum and retention time as an authentic standard of (–)-α-Bisabolol on a chiral column (**Figure 4**). To confirm the stereo selectivity of EeTPS1 we also injected extracts from *L. dulcis* [(+)-epi-α-bisabolol; Attia et al., 2012] and *Artemisia* [(+)-bisabolol; Muangphrom et al., 2016]. The three stereo isomers are clearly separated. Thus, EeTPS1 was identified as an (–)-α-Bisabolol synthase, and was renamed to EeBOS.

The EeBOS protein sequence was aligned to the protein sequences of the currently known (–)-α-bisabolol synthase of *M. recutita* (Son et al., 2014), the (+)-epi-bisabolol synthase from *L. dulcis* (Attia et al., 2012), and the α-bisabolol synthases from *A. annua* (Li et al., 2013), (+)-α-bisabolol synthases from *Artemisia kurramensis* and *Artemisia maritima* (Muangphrom et al., 2016; **Supplementary Figure 2**). The EeBOS protein sequence displayed 71% identity to the *Matricaria* synthase (MrBOS), but only 48% identity to the *Artemisia* synthases, and 35% identity to the synthase of *L. dulcis* (**Supplementary Table 3**). Both the *Matricaria* and Candeia amino acid sequences showed an elongated N-terminus, compared to the other proteins (**Supplementary Figure 2**). Interestingly we could only detect (–)-α-bisabolol as the product of EeBOS, whereas the MrBOS was also producing a small (2% of the total terpenoids) quantity of β-farnesene (Son et al., 2014).

### Gene Expression Analysis of EeBOS1

**FIGURE 5:**
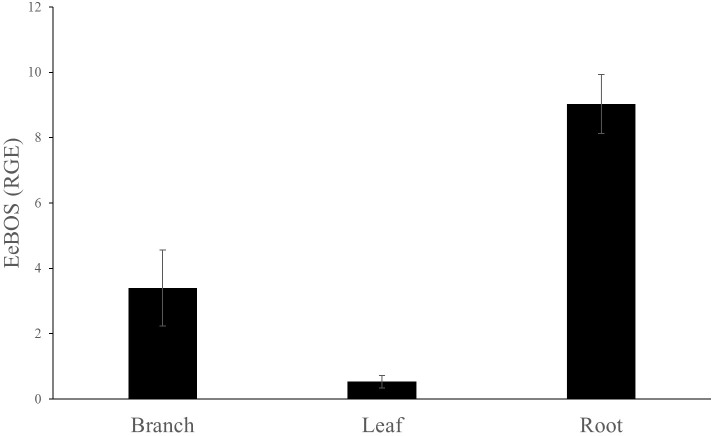
Relative gene expression of EeBOS in different tissues. The reference gene used was EeEF1. Each bar represents the average of at least three biological replicates ± SE.

In order to correlate the expression of the EeBOS to the presence of (–)-α-bisabolol, samples from the branch, leaves and roots of the trees were analyzed for gene expression with quantitative RT-PCR (**Figure 5** and **Supplementary Figure 4**). Specific primers were designed based on the Candeia cDNA sequence (**Supplementary Table 2**). The reference gene used was the Candeia elongation 1-alpha factor (EeEF1).

EeBOS expression followed the (–)-α-bisabolol accumulation pattern (**Figure 5** and **Supplementary Figure 4**). Interestingly, low levels of expression of EeBOS1 could be detected in leave samples whereas no bisabolol could be detected in these samples (**Figure 3**). Otherwise, levels of gene expression of EeBOS parallel the bisabolol levels in the analyzed tissues, confirming a role of the EeBOS gene in production of (–)-α-bisabolol, and suggesting that regulation of its expression contributes to the control of the quantity of (–)-α-bisabolol in the Candeia tree.

## Discussion

In this work, we identify EeBOS, a gene which appears to be able to mediate the synthesis of the broadly used compound (–)-α-bisabolol, from the Candeia tree. This is the first step in the elucidation of the pathway to bisabolol in this important plant. On the longer term, EeBOS may provide an important tool for the breeding of highly productive Candeia germplasm, which is needed for the sustainable production of (–)-α-bisabolol and the preservation of the wild Candeia resources. The observation that (–)-α-bisabolol is also produced in the root of the Candeia tree could suggest that alternative ways of (–)-α-bisabolol using hairy root cultures could be explored, as an alternative for commercial plantations.

## Author Contributions

JB, KC, CC, and HB contributed conception and design of the study. LAGA, TD, and KC generated the data. AvH, FV, and AGB performed the chemical analysis and sampling. LAGA and TD wrote the first draft of the manuscript. JB and FV wrote sections of the manuscript. All authors contributed to manuscript revision, read, and approved the submitted version.

## Conflict of Interest Statement

The authors declare that the research was conducted in the absence of any commercial or financial relationships that could be construed as a potential conflict of interest.
