# Supplementary material for: Identification of the Bisabolol Synthase in the Endangered Candeia Tree (Eremanthus erythropappus (DC) McLeisch)
Source: Front Plant Sci. 2018 Sep 19;9:1340. doi: 10.3389/fpls.2018.01340 (PMC6158398; doi:10.3389/fpls.2018.01340)
Supplement: Supplementary file 1 [file Presentation_1.pdf]

Supplementary Material

Identification of the bisabolol synthase in the endangered Candeia tree (Eremanthus erythropappus (DC) McLeisch)

Leticia Alves Gomes Albertti, Thierry L. Delatte, Katyuce Sousa de Farias, Amanda Galdi Boaretto, Francel Verstappen, Adele van Houwelingen, Katarina Cankar, Carlos Alexandre Carollo, Harro J. Bouwmeester, Jules Beekwilder

Corresponding author
Jules Beekwilder
jules.beekwilder@wur.nl

Supplementary table 1. Main components of Candeia tissues, at three development stages.

RT	RI	Compound	Sapling (<1 year)			5 year old tree							10 year old tree										Identification method
			Leaf	Stem	Root	Twig	Limb	Leaf	New root	Root	Inflorescence	Seeds	Twig	Limb wood	Limb Bark	New leaf	Old leaf	New root	Old root	Old root core	Trunk core	Trunk bark	
11.80	1336	Elemene	1.20	-	-	-	-	-	0.23	-	-	-	-	-	-	-	-	-	-	-	-	-	RI, MS
11.91	1342	Presilphiperfol-7-ene	-	-	0.38	-	-	-	3.28	14.98	-	-	-	-	-	-	-	-	-	-	-	-	RI, MS
12.07	1348	α-Cubebebene	-	-	0.14	-	-	-	0.70	-	-	-	-	-	-	0.22	0.37	-	-	-	-	-	RI, MS
12.13	1350	Silphin-1-ene	-	-	0.14	-	-	-	1.17	-	-	-	-	-	-	-	-	-	-	-	-	-	RI, MS
12.44	1370	Cyclosativene	-	-	0.24	-	-	-	2.70	-	-	-	-	-	-	0.45	-	-	-	-	-	-	RI, MS
12.40	1379	α-Copaene	30.61	-	1.46	24.17	-	16.17	2.23	-	15.37	-	1.54	-	-	23.41	23.40	-	-	-	-	-	RI, MS
12.60	1389	β-Elemene	4.65	-	1.27	-	-	1.92	1.64	-	1.51	6.98	-	0.12	-	1.79	2.46	0.25	-	0.23	-	-	RI, MS
12.72	1388	α-Isocomene	-	-	7.93	-	-	-	0.70	-	-	-	-	-	-	-	-	-	-	-	-	-	RI, MS
12.79	1399	sesquithujene	-	-	0.28	-	-	-	0.47	-	-	-	-	-	-	-	-	-	-	-	-	-	RI, MS
12.91	1402	cyperene	-	-	9.87	-	-	-	10.20	14.48	-	21.85	-	-	-	-	-	0.12	-	-	-	-	RI, MS
12.93	1413	α-Gurjunene	1.80	-	-	-	-	-	-	-	2.41	-	-	-	-	2.02	1.97	-	-	-	-	-	RI, MS
13.10	1420	(E)-β-Caryophyllene	28.03	68.90	7.40	34.49	-	28.19	4.30	-	27.14	-	1.34	-	-	23.53	24.63	0.12	-	-	-	-	RI, MS, ST
13.45	1437	Sesquisabinene-A	0.68	-	3.90	-	-	-	2.26	-	-	-	-	-	-	-	-	-	-	-	-	-	RI, MS
13.50	1440	Humulene	3.94	-	1.49	-	-	4.27	2.27	-	3.87	-	-	-	-	3.36	2.83	-	-	-	-	-	RI, MS
13.88	1480	Germacrene D	10.72	31.10	1.99	-	-	25.53	2.11	-	23.44	-	1.77	-	-	20.17	19.11	-	-	-	-	-	RI, MS
14.03	1485	β-Selinene	-	-	13.63	-	-	-	1.88	-	-	-	-	-	-	-	-	-	-	0.58	-	-	RI, MS
14.06	1494	Bicyclogermacrene	4.41	-	1.92	-	-	12.71	-	-	11.50	-	-	-	-	10.64	9.85	-	-	0.58	-	-	RI, MS
14.19	1496	α-Bisabolene	-	-	29.83	-	-	-	-	-	-	-	-	-	-	-	-	-	-	0.58	-	-	RI, MS
14.30	1513	Amorphene (γ-cadinene)	4.52	-	3.22	-	-	11.21	4.40	-	9.46	20.27	-	-	-	11.27	9.85	-	-	-	-	-	RI, MS
15.13	1570	Caryophyllene oxide	1.07	-	3.97	-	-	-	-	-	1.08	-	-	-	-	-	-	-	-	-	-	-	RI, MS
15.30	1592	Presilphiperfolanol	-	-	1.93	-	-	-	24.14	31.11	-	-	-	-	-	-	-	-	-	-	-	-	RI, MS
15.70	1610	Cubenol	-	-	0.46	-	-	-	8.44	-	1.08	-	-	-	-	-	-	-	-	-	-	-	RI, MS
15.80	1636	Cadinol	2.97	-	6.55	-	-	-	-	-	-	42.79	-	-	-	1.57	3.08	-	-	-	-	-	RI, MS
15.90	1652	α-Cadinol	2.81	-	-	-	-	-	3.75	-	3.20	8.11	-	-	-	1.62	2.46	-	0.60	-	-	-	RI, MS
15.96	1744	Bisabololoxide	-	-	-	-	-	-	-	-	-	-	-	-	-	-	-	0.62	0.60	1.97	-	-	RI, MS
16.27	1683	(−)-α-bisabolol	-	-	-	26.11	36.59	-	0.59	5.71	-	-	32.31	76.92	0.43	-	-	37.45	44.18	39.35	30.23	-	RI, MS, ST
19.05		sesquiterpene lactone (Vanillosmin)	2.58	-	0.31	-	-	-	-	-	-	-	-	-	49.79	-	-	5.26	7.88	35.88	46.51	35.50	MS
19.17		Diterpene I	-	-	0.99	15.23	23.65	-	2.34	17.55	-	-	1.54	-	1.42	-	-	-	0.17	-	-	-	MS
19.68		Diterpene II	-	-	0.66	-	39.76	-	20.16	16.17	-	-	61.54	22.96	48.36	-	-	56.18	46.57	20.83	23.26	64.50	MS

Supplementary table 2. Primers use during this study

Q-PCR_EeBOS	Fw	CGGGAAATTCGATTCTGGAG
Q-PCR_EeBOS	Rv	CAAGCACCCAGGCATATT
Q-PCR_EeEF1	Fw	GGCTGATTGTGCTGTTCTTATC
Q-PCR_EeEF1	Rv	ACCCCAAGAGTGAAAGCAAG
Chrysolaena EF	Fw	CTGGAGGAATTGTACAAG
Chrysolaena EF	Rv	GCTTGACCCCAAGAGTGA
EeBOS_NotI	Rv	ATATGCGGCCGCTCAAACAACTAAAGGGTGAACAACGAGC
EeBOS_SalI	Fw	ATATGTCGACCGATCAAATCATGTCAACTGCTTTTCCA
RACE primer		CAGTGTAGTGCATTATCAGTGCGAGTG

Supplementary table 3. Amino acid identity (%) of six Bisabolol synthase

		1	2	3	4	5	6
PdBOS	1	-	32.2	32.02	31.83	35.27	35.2
AmBOS	2	32.2	-	98.53	94.14	48.05	48.35
AkBOS	3	32.02	98.53	-	94.14	48.24	48.53
AaBOS	4	31.83	94.14	94.14	-	48.05	48.53
EeBOS	5	35.27	48.05	48.24	48.05	-	70.85
MrBOS	6	35.2	48.35	48.53	48.53	70.85	-

AkBOS = *Artemisia kurramensis* bisabolol synthase (BAW34955.1); AaBOS= *Artemisia annua* bisabolol synthase (AFV40969.1); AmBOS= *Artemisia maritima* bisabolol synthase (BAW34954.1); McBOS= *Matricaria recutita* bisabolol synthase (AIG92846.1); PdBOS= *Phyla dulcis* bisabolol synthase (AFR23372.1)

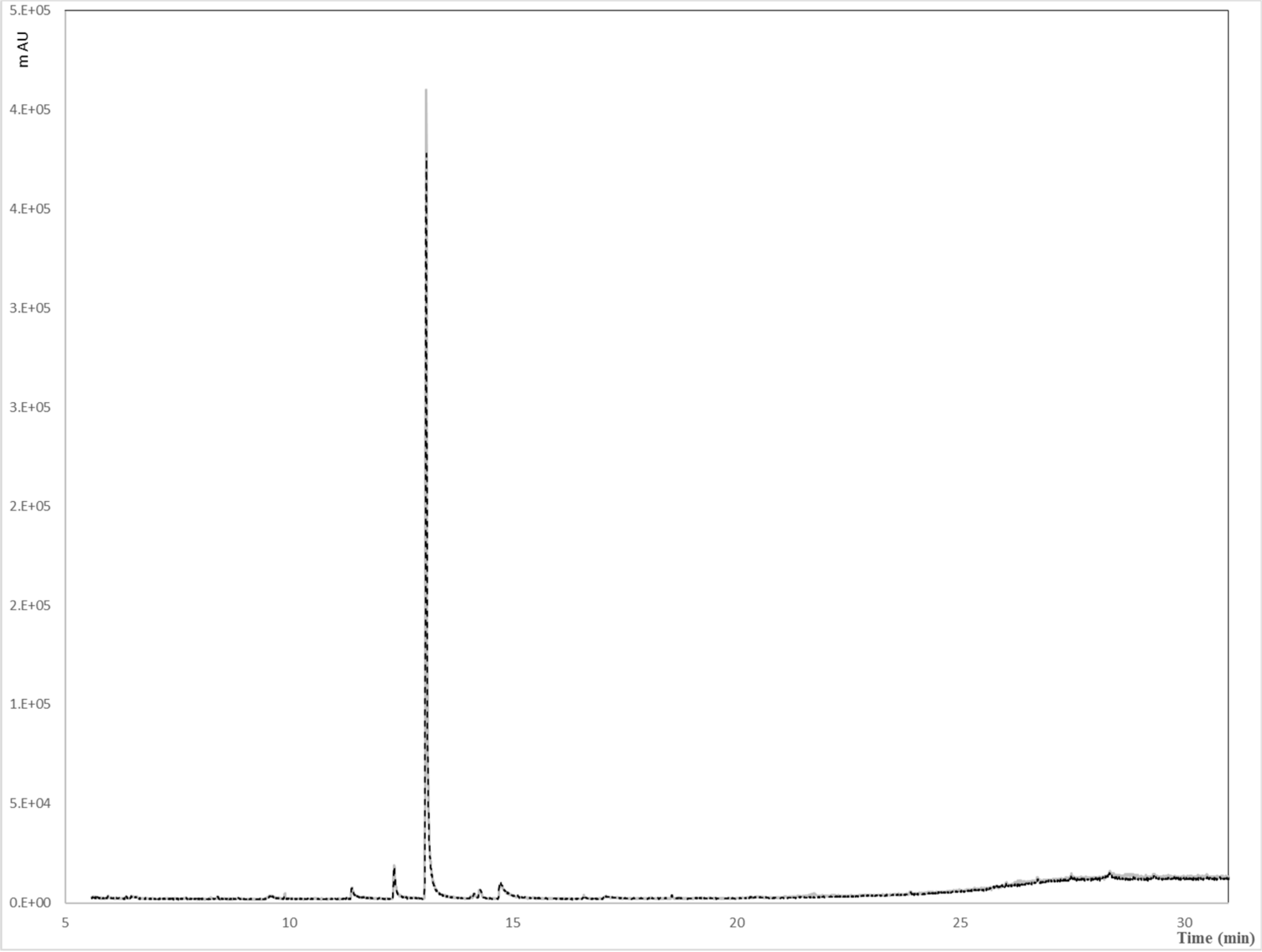

Supplementary figure 1. Detection of *in vitro* EeBOS products with GPP as a substrate. In vitro enzymatic assay with cell extracts expressing (black dotted line) or not (grey solid line) EeBOS were compared in the presence of GPP. The results were analysed by GC-MS.

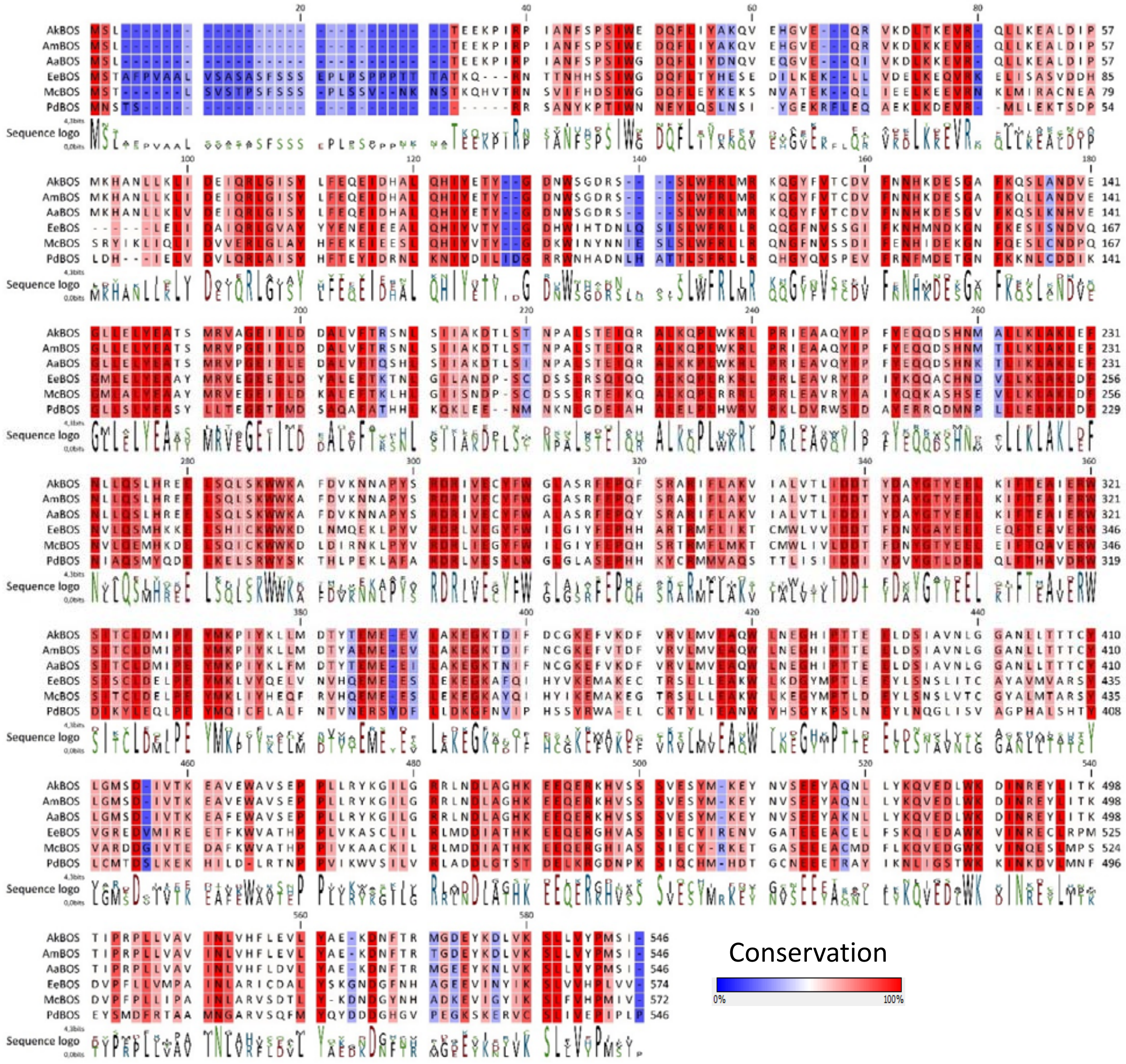

Supplementary figure 2. Protein alinement between six bisabolol synthase. The conservation of amino acids in the protein are depicted with a rage from low to highly conserved (from blue to red, respectively). AkBOS = *Artemisia kurramensis* bisabolol synthase (BAW34955.1); AaBOS= *Artemisia annua* bisabolol synthase (AFV40969.1); AmBOS= *Artemisia maritima* bisabolol synthase (BAW34954.1); McBOS= *Matricaria Recutita* bisabolol synthase (AIG92846.1); PdBOS= *Phyla dulcis* bisabolol synthase (AFR23372.1)

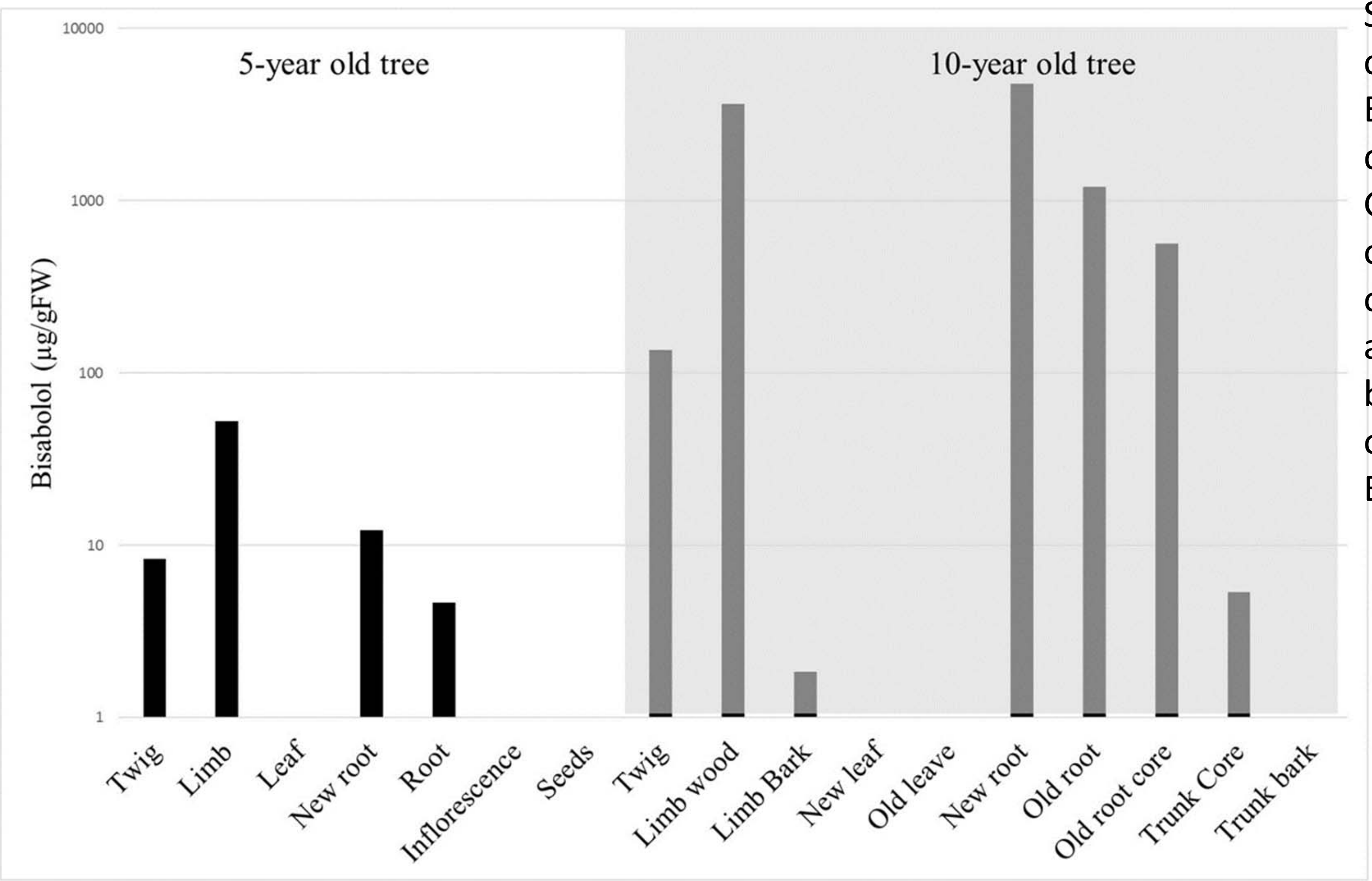

Supplementary figure 3. Bisabolol content in different tissues. (−)-α-Bisabolol was extracted with dichloromethane and quantified by GC-MS for different tissues originating from the 5 and 10-year old trees. Each bar represents a single analysis. The grayed out area of the bar diagram represent sample originating from the 10-year old tree. Each bar represents a single analysis.

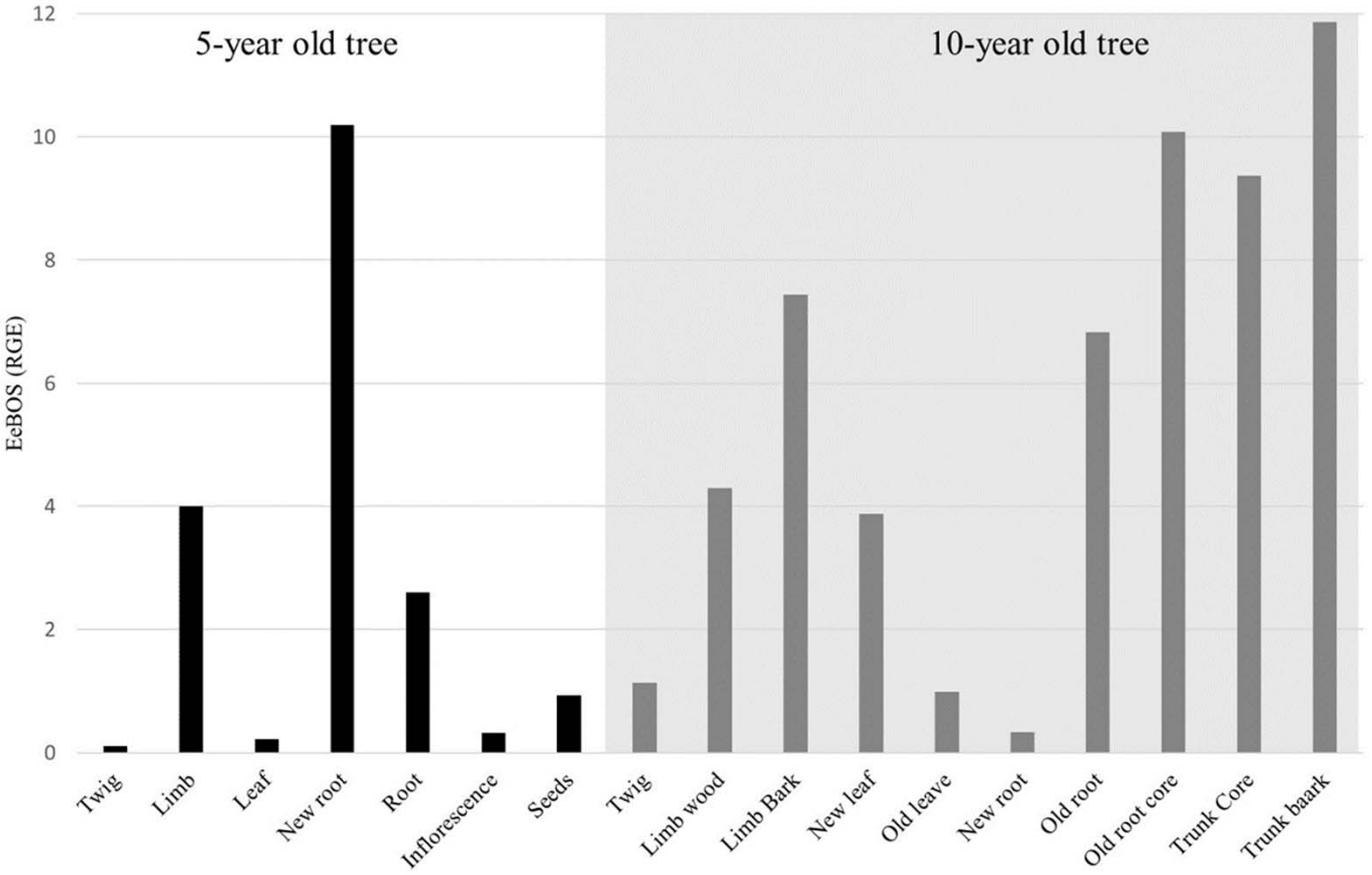

Supplementary figure 4. Relative gene expression of EeBOS in different tissues. The reference gene used was EeEF1. The grayed out area of the bar diagram represent sample originating from the 10-year old tree. Each bar represents a single analysis.
